# Helminths in the gastrointestinal tract as modulators of immunity and pathology

**DOI:** 10.1152/ajpgi.00024.2017

**Published:** 2017-03-16

**Authors:** Fumi Varyani, John O. Fleming, Rick M. Maizels

**Affiliations:** ^1^Wellcome Centre for Molecular Parasitology, Institute of Infection, Immunity and Inflammation, University of Glasgow, Glasgow, United Kingdom;; ^2^Edinburgh Clinical Academic Track, Western General Hospital, University of Edinburgh, Edinburgh, United Kingdom; and; ^3^Department of Neurology, University of Wisconsin School of Medicine and Public Health, Madison, Wisconsin

## Abstract

Helminth parasites are highly prevalent in many low- and middle-income countries, in which inflammatory bowel disease and other immunopathologies are less frequent than in the developed world. Many of the most common helminths establish themselves in the gastrointestinal tract and can exert counter-inflammatory influences on the host immune system. For these reasons, interest has arisen as to how parasites may ameliorate intestinal inflammation and whether these organisms, or products they release, could offer future therapies for immune disorders. In this review, we discuss interactions between helminth parasites and the mucosal immune system, as well as the progress being made toward identifying mechanisms and molecular mediators through which it may be possible to attenuate pathology in the intestinal tract.

helminth infections are highly prevalent in most tropical and developing countries, yet notably, these areas also suffer relatively low levels of “diseases of modernity” associated with hyperactive immune responsiveness (105, 181). While economic development has reduced or eliminated helminth infections, there has been an inexorable rise in the incidence of immunological disorders such as allergy, autoimmunity, and inflammatory bowel disease. One possible explanation is that helminths (and the immunomodulatory molecules they produce) directly modulate the host immune system to attenuate development of antiparasite immunity, in a manner that may also dampen bystander immune pathologies (104, 116).

Helminths are multicellular worm parasites that have evolved to occupy a vast range of niches, including the gastrointestinal tract of vertebrate hosts (Table 1). In general, they establish long-lived, chronic infections characterized by widespread downmodulation of both the innate and adaptive arms of host immunity. Hence, the presence of intestinal helminths may block the same inflammatory pathways that are responsible for allergies and autoimmunity, raising the potential for novel therapies based on the molecules and/or the pathways that parasites have evolved to suppress host immune reactions (51, 69, 113).

**Table 1.** Major helminth parasites including species implicated in modulating colitis

Phylum	Species	Notes
Cestodes (Tapeworms)	*Echinococcus granulosus*	Causes hydatid cysts of the liver following ingestion of eggs from dogs.
	*Hymenolepis diminuta*	Small tapeworm of rats; other members of genus can infect humans.
	*Taenia saginata, T. solium*	Human tapeworms, transmitted through undercooked beef or pork; can cause cysticercosis and neurocysticercosis.
Nematodes (Roundworms)	*Ancylostoma caninum, A. ceylanicum, A. duodenale*	Hookworms of dogs and humans, larvae in soil penetrate skin and home to gut via the lungs.
	*Anisakis simplex*	Parasite of marine mammals; larvae in fish can infect humans if eaten raw.
	*Ascaris lumbricoides*	Common roundworm of human; infects ~800 million people; direct fecal-oral transmission through eggs in environment.
	*Brugia malayi*	Lymphatic filarial parasite, mosquito-borne, causes elephantiasis
	*Heligmosomoides polygyrus*	Mouse intestinal nematode related to hookworm, widely used model system.
	*Necator americanus*	Human hookworm; together with *A. duodenale* infects ~600 million people.
	*Strongyloides stercoralis*	Threadworm, infects intestinal tract and causes strongyloidiasis. Can autoinfect the host, hence lifelong infection.
	*Toxascaris leonina*	Large roundworm of cats and canids, closely related to *Ascaris* in humans.
	*Trichinella spiralis*	Pork worm, contracted from undercooked meat, larvae invade muscle cells of the host.
	*Trichuris trichiura*	Whipworm in large intestine; infects ~600 million people. Related species from pigs (*T. suis*) used in helminth therapy.
	*Wuchereria bancrofti*	Lymphatic filarial parasite, mosquito-borne, causes elephantiasis.
Trematodes (Flukes)	*Clonorchis sinensis*	Liver fluke prevalent in Asia, can cause cholangiocarcinoma.
	*Schistosoma japonicum*	Causes schistosomiasis japonica, hepatosplenic disease; transmitted through intermediate snail host releasing water-borne invasive cercarial larvae.
	*Schistosoma mansoni*	Widespread cause of schistosomiasis, together with *S. hematobium* and *S. japonicum*, afflicting ~200 million people.

Even today, helminth infections affect around one quarter of people in the world (74, 140) and in historic times would have been near universal in the human population, so that these parasites have been long-term companions acting to shape the immune system. Indeed, helminth parasitism of the vertebrate gastrointestinal tract has been noted in fossils dating to the early Cretaceous period, ~125 million years ago (MYA) (138); additionally, the ubiquitous presence of geohelminths, such as the genus *Trichuris*, in many animal species suggests that parasite coevolution paralleled the mammalian adaptive radiation, starting 65 MYA. In fact, gastrointestinal helminth parasitism is likely present in virtually every mammal residing in a “natural” habitat.

Obviously, some parasitic species, especially those of relatively recent introduction to humans, are a major public health scourge and cause significant morbidity and mortality worldwide (75). On the other hand, the long coevolutionary history of helminths and their hosts has resulted in many parasites being relatively well tolerated and even contributing through their subtle dampening of inflammation to an optimal immunological balance (1). Thus, in modern times, the absence of helminths may lead to the immune system “overshooting” and mounting deleterious responses to harmless environmental and self-antigens.

Importantly, in many instances, a host’s environment includes external and endogenous microbes, which must be tolerated or even accepted as beneficial. In immunological terms, there is a continuum from commensal microbes through to the “macrobionts,” such as helminths (55). Across this entire “multibiome” (49), wherever pathogenic consequences are minimal, an immunological equilibrium or truce is adaptive for both parasite and host; thus, to promote its own survival in the host during a chronic infection, a parasite may limit pathology, which significantly affects the host’s fitness, and to avoid serious collateral damage to its own tissues, a host may attenuate its immune responses to the parasite.

At present, there is increasing molecular definition of how microbes contribute to healthy immunological homeostasis in the gut (3, 49, 73, 147). In what follows, we will provide evidence that demonstrates that certain helminth species may similarly restrain excessive reactivity of the mucosal immune system, often in a highly directed manner (43, 71, 160). These findings have led to the currently intensifying interest in helminth-derived agents as potential new therapeutic tools for allergic, autoimmune, and inflammatory bowel diseases (44, 114, 126, 137).

## Helminths and the Hygiene Hypothesis

In 1989, in an epidemiological survey of family size and birth order in British school children with hay fever and eczema, Strachan (165) found that the prevalence of both of these conditions was reduced in younger siblings within larger families. Strachan proposed that this protective effect might be due to early childhood infections, a supposition which later evolved into various forms of the “hygiene hypothesis” (11, 101, 164, 178, 185). These and many other authors have significantly elaborated on the hygiene hypothesis concept, first by encompassing the full range of allergic and autoimmune conditions, asthma, type 1 diabetes, rheumatoid arthritis, ulcerative colitis, Crohn’s disease, and multiple sclerosis, to consider the upsurge in inflammatory disorders in the developed world (11, 101, 185). Second, early forms of the hygiene hypothesis proposed that early life microbial infections protected against allergy by promoting Th1-type responses at the expense of the proallergic Th2 arm of immunity, which mediates allergy. However, most nonallergic inflammatory conditions are themselves Th1 (and/or Th17) mediated, arguing against a simple Th1/17 vs. Th2 seesaw determining inflammatory status. With the recognition that eukaryotic parasites are also very effective at dampening immunological reactivity of their host through regulatory T-cell (Treg) expansion (101, 183), the hygiene hypothesis expanded to evoke immunosuppressive regulatory cells as a key pathway by which infectious agents could impact on the control of allergies and autoimmunity (50, 102).

Further significant reformulations of the hygiene hypothesis include the “Old Friends hypothesis” (146), which emphasizes protection provided by evolutionary ancient commensal and environmental microbiota, as well as the “Microflora hypothesis” (129, 148), which focuses on the role of gut bacteria in shaping systemic immune responses and extends the role of dietary metabolites (171), and finally, the “Biodiversity hypothesis” (67), which underscores potential health effects in a biosphere impacted by loss of biodiversity and by climate change. Bringing all this together, Filyk and Osborne (49) have introduced the term “multibiome” to comprehensively describe the bacteria, viruses, fungi, and multicellular organisms, which together colonize the gastrointestinal system and influence immune homeostasis in health and disease. Thus, while helminth parasites share the host environment with multiple other forms of life, it is notable that numerous epidemiological, animal model, and clinical investigations have identified a prominent role of helminths in putative protection from allergy and autoimmunity, often linked to the regulatory arm of the immune system (59, 68, 105, 161). It is interesting to note that Tregs are also implicated in many studies of the microbiota’s influence on host immunity (57). In particular, *Bacteroides fragilis* expresses polysaccharide A, which induces Tregs to protect mice from colitis (149). Similarly, species of *Bifidobacterium* (131), *Clostridium* (8, 9), and *Lactobacillus* (83) have all been shown to induce Tregs in the gut, which are important in creating a stable anti-inflammatory environment (145). Failure or an imbalance in this process may result in pathology, most notably, inflammatory bowel disease (IBD) (13).

The association between parasite infection and reduced prevalence of immune disorders was first noted by Greenwood (62) in 1968 with respect to rheumatoid arthritis in African populations with high endemic helminth exposure. Subsequently, the first clear evidence of the role of parasitic infections in modulating allergy came from studies on Gabonese school children in an area endemic for schistosomiasis; infected children had lower reactivity (measured by skin prick testing) than uninfected contemporaries (174); moreover, when infected children were given antihelminth therapy, they showed an increase in mite skin test positivity (175). Similar data linking helminth infections with attenuated allergy have been reported in South American populations by independent investigators (5, 28).

Helminths may also modulate many other inflammatory and autoimmune conditions in humans. A series of reports on multiple sclerosis patients in Argentina linked remission of disease with acquisition of gastrointestinal helminth infections (29) and found disease relapses following clearance of parasites in a subset of these patients (30). In a population-based study in Zimbabwe, schistosome-infected subjects bore lower levels of circulating autoimmune antinuclear antibody, which increased significantly following antischistosome therapy (125). Finally, with respect to inflammatory bowel diseases, there are both case reports (17) and small-scale trials indicating that helminth infections can confer a protective effect on patients (44, 181).

The original hygiene hypothesis focused on early life imprinting of the immune system by environmental exposure to microbes; however, helminths may similarly exert lifelong effects. Parasite-specific tolerance was induced in children of mothers exposed to the filarial nematode parasite *Wuchereria bancrofti* in pregnancy (163). Early life exposure to helminths also modulates responses to allergens, as shown by a study in which antihelminthic treatment of pregnant mothers resulted in a higher incidence of atopic eczema in infants than in those born to untreated infected mothers (124). Furthermore, childhood exposure to helminths was found to be protective against both Crohn’s disease and ulcerative colitis (24).

This fascinating interaction between environmental imprinting during infection and the known genetic predisposition of humans to inflammatory diseases (155) raises an interesting question of mechanism, which may be answered by arena field of epigenetics. Epigenetics refers to stable and inheritable alterations in gene expression without altering the DNA nucleotide sequence but through chemical modification of DNA bases (e.g., methylation) and DNA-associated histone proteins (by methylation and acetylation) (7). Prime examples of plasticity following environmental challenge are epigenetic alterations in innate immune cells, such as macrophages (151), as well as activated effector and T lymphocytes (182). Indeed, reports are already emerging on epigenetic control of the response to helminth parasites (21, 27, 72), as well as in a range of inflammatory diseases (7, 98, 132), suggesting that epigenetic research will provide a strong theoretical and empirical basis for understanding the modulatory effects of helminths in the gastrointestinal tract during autoimmunity and allergy.

The increase in immunological reactivity following antihelminthic clearance demonstrates, however, that the immune system is not always immutably imprinted by parasite exposure, but responsive to its current infection status. In fact, helminth infection in later life can very clearly downmodulate immune hyperactivity (104, 116, 181), leading as discussed below to trials using live parasites to treat inflammatory conditions such as IBD (168) and celiac disease (51, 112).

## Helminths and the Immune System

Helminth parasites encompass a myriad of different life histories with particular dynamics and properties, which drive a wide diversity of immune responses (Table 1). Together with multiple environmental variables (coinfections, comorbidities, diet, and climate) and polymorphisms in host immune response genes, it is not surprising that different helminth infections may either exacerbate or ameliorate allergy and autoimmunity (111, 153, 161), and consideration of immune modulation by helminths must take these other factors into account.

In humans and livestock, intestinal helminths include the nematode roundworms and the cestode tapeworms. Each species possesses a particular migratory cycle and tropism and generally localizes to a specialized anatomical niche. For example, schistosomes, hookworms, and *Strongyloides* larvae penetrate unbroken skin and travel to the lung before migrating either to the mesenteric vasculature or the lumen of the gut. Other parasites, such as immature stages of tapeworms and the nematode *Trichinella*, leave the gut to encyst in muscle for transmission to a new carnivorous host. Such helminths can cause severe inflammation as in the case of schistosome trematodes, releasing eggs that either transit through the intestinal wall or lodge in the liver causing fibrosis (16, 48). However, apart from the blood-feeding hookworms, many of the parasites that establish in the intestinal lumen are not directly pathogenic to their surrounding tissue.

The immune response to helminths is generally dominated by the type 2/Th2 pathway that serves to directly trap, kill, or expel parasites, alongside an expanded Treg compartment that modulates and dampens inflammation (63, 100). This creates an environment in which helminths cannot thrive while also promoting repair of the physical damage caused by the worms (1, 54) and is in contrast to the classical inflammatory type 1 response targeted at bacterial and viral microorganisms.

The type 2 response is principally effected through the IL-4Rα and STAT6 pathways (1, 173), driven by either or both IL-4 and IL-13. In helminth infections, type 2 immunity is initiated at the site of invasion by epithelial cells, which release the alarmins IL-25 and IL-33, inducing innate lymphoid cells (ILCs) to produce IL-13 and other cytokines. In the absence of either IL-25 or IL-33, resistance to helminth infections is severely impaired (127), as is the case in IL-4Rα or STAT6 deficiency (173).

The IL-4Rα-dependent adaptive immune response includes antigen-specific Th2 lymphocytes that produce cytokines IL-4, IL-5, IL-9, and IL-13 (176), and type 2 phenotype (M2) alternatively activated macrophages (90). Type 2 macrophages are centrally involved in the antihelminth response and repair mechanisms through molecules such as arginase-1, TIMP1 and -2 (inhibitors of metalloproteases), and IGF-1, which promotes fibroblasts and myofibroblast matrix formation (2, 90).

Tregs police the immune system to prevent untoward inflammatory reactions against self-antigens and innocuous environmental substances, while also terminating responses to pathogens when no longer required (152). They characteristically express the transcription factor Forkhead box P3 (Foxp3) and suppress both effector Th1 and Th2 cells through both direct cell surface interactions and by the secretion of TGF-β and IL-10. A defect in the Foxp3 gene results in fatal autoimmunity in mice and the IPEX syndrome in humans (immune dysregulation, polyendocrinopathy, enteropathy, X-linked syndrome) with extensive inflammation, particularly in the gastrointestinal tract (10). Tregs have a dual role in helminth infections: they protect the host from excessive inflammatory response to infection, but they also may reduce protective immunity and, thereby, permit infections to establish chronicity (34, 154, 159, 170). Reflecting the dependence of helminths on the regulatory compartment, it has been found that some helminths are able to induce the development of Tregs to modulate the immune response (61, 186).

It is important to recognize also that the immune response to helminth infection may evolve dramatically over time, following developmental changes in parasite migration or maturation, and/or time-dependent switches in immune activation or regulation. A classic example is in schistosomiasis, in which an initial Th1 response is superseded by a dominant Th2 mode once parasite egg release has commenced (135). Similarly, Nutman (130) and Santiago and Nutman (153) have mapped the evolution of a typical immune response to helminths, from the initiation of infection at mucosal surfaces, when a broad and robust inflammation, primarily mediated by effector Th1, Th2, and Th17 CD4^+^ cells, attempts to abort the infection; if unsuccessful, a period of weeks or months following, during subacute or latent infection is characterized by a more limited or focused Th2 reaction, primarily mediated by Th2 CD4^+^ cells, IL-4, IL-5, and eosinophils, which together minimize parasitic load. If a chronic infection is established over the succeeding months or years, the host response becomes essentially immunomodulatory and is primarily mediated by regulatory cells (1, 44, 50, 161) and anti-inflammatory cytokines (e.g., IL-10 and TGF-β) to assure that low levels of helminths are tolerated and immune homeostasis prevails. While this vignette is, of course, oversimplified, it well illustrates the alternative modes of antihelminth immune responsiveness and is important in considering whether immune modulation is differentially evoked during different phases of infection (45, 97).

## Immune Mechanisms in the Gastrointestinal Tract

The intestine is the crucial barrier surface that must both obtain nutrition and protect the host. In this milieu, the immune system is constantly exposed to pathogens and foreign antigens, and its cells must discriminate pathogenic from harmless stimuli to mount protective responses, while maintaining homeostasis by tolerating food antigens, nonpathogenic bacteria, and helminths (79, 136). In addition, the immune system must compensate for the effects of the pathogen, reducing both the damage caused by the pathogen itself and the collateral immunity-mediated damage necessary to clear the invading organism (22).

The epithelial cells of the intestine, which are the first responders to gut infection, consist of the enterocytes, goblet cells, neuroendocrine cells, Paneth cells, and tuft cells. Together, the intestinal epithelial cells perform an essential barrier role, including intercellular tight junctions, which prevent pathogens from breaching the GI tract (6). The epithelial cells express pattern recognition receptors, such as Toll-like receptors and nucleotide-binding oligomerization domain-like receptors to sense pathogenic bacterial products such as LPS. Epithelial cells also respond to physical invasion and trauma by releasing alarmin cytokines that stimulate innate lymphoid and dendritic cells to initiate an immune response.

Distributed along the small intestinal epithelium, particularly in the more distal ileum, are lymphoid aggregates known as Peyer’s patches (82). Each patch is surrounded by follicle-associated epithelium, which consists of follicle-associated enterocytes and M cells that sample the surrounding microenvironment. M cells and other specialized cells beneath the epithelial barrier generate the antigen-specific response necessary for antibody production and generation of immunological memory. M cells have microfolds instead of microvilli and a basolateral pocket containing T and B lymphocytes, macrophages, and dendritic cells (92). Activated dendritic cells travel via the lymphatics to the gut-draining mesenteric lymph nodes, where they present antigens to naïve T cells and coordinate adaptive responses (64).

Interestingly in helminth infections, three specialized epithelial cell subtypes are prominent: the goblet cells, Paneth cells, and tuft cells. Goblet cells secrete mucins, trefoil peptides, and resistin-like molecules, which make up mucus (88). These are secreted by exocytosis in response to external stimuli, such as microbes, cytokines, and inflammation. The mucus functions as a lubricant and helps maintain the barrier between the epithelium and the intestinal microbiota (109). Paneth cells are present at the base of crypts in the small intestine and play a dual role in nourishing adjacent intestinal stem cells and releasing important antimicrobial molecules (25), including lysozyme, phospholipase A2, and antimicrobial defensins. Very recently, a little-studied epithelial cell type, the tuft cell, has been discovered to play a major role in antihelminth immunity, through the production of the alarmin IL-25 (56, 76, 177). Mice lacking the transcription factor required for tuft cell differentiation, Pou2f3, are devoid of Tuft cells and unable to expel intestinal helminths unless exogenous IL-25 is administered (56).

In intestinal helminth infection, alarmin release and production of Th2 cytokines stimulate muscle peristalsis and epithelial fluid egress, constituting a “weep and sweep” model for helminth expulsion. As well as goblet cell mucus release, mast cell proteases degrade tight junctions and allow intestinal fluids to leak into the intestinal lumen (110), and the smooth muscle contracts to effectively sweep the helminths away (4, 99, 103). In addition, epithelial cells increase their rate of turnover to produce an “epithelial escalator” to expel the helminth (26).

## Inflammatory Bowel Diseases

Ulcerative colitis (UC) and Crohn’s disease (CD) are both IBDs that result in significant long-term morbidity and mortality (118). CD results in predominantly gastrointestinal symptoms, including abdominal pain, fever, and diarrhea with blood and mucus (14). The disease can manifest anywhere along the GI tract and can also result in nongastrointestinal features such as uveitis and enteropathic arthritis. UC affects the colonic mucosa and predominantly presents with bloody diarrhea (134), and also differs immunologically from CD in displaying an atypical Th2-like inflammatory condition (35).

Celiac disease is an autoimmune gluten-sensitive small-intestinal enteropathy triggered by gluten in cereals (123, 162). This can present with diarrhea, abdominal pain, distension, and vitamin deficiency, as well as failure to thrive in children. Celiac disease is treated by consuming a gluten-free diet; however, there are cases of refractory disease that may benefit from immunomodulatory therapies.

IBD is accompanied by a high level of T-cell cytokine production, in particular, expansion of inflammatory Th1 cells; under control of the transcription factor Tbet, Th1 cells produce IFN-γ and TNF in response to appropriate costimulatory signals from gut antigen-presenting dendritic cells (DCs) and macrophages. In experimental mouse models of IBD, the effect of regulatory T cells is decisive in determining disease progression. In mice lacking T and B cells, [for example, SCID or recombination-activating gene (RAG) deficient], the lymphocyte compartment can be reconstituted by the transfer of syngeneic cells from wild-type donors. However, if regulatory T cells are depleted from the transferred population, the remaining CD4^+^ effector T-cell populations cause a chronic colitis with a Th1 pattern of cytokine synthesis (IFN-γ and TNF) (106, 139).

IBD-like colitis can also be generated by stimulating innate cells in RAG-deficient mice with anti-CD40 activating antibodies (172) or by causing gross epithelial damage with agents, such as dextran sodium sulfate (DSS) (133). Blocking TNF reduces the severity of DSS colitis in mouse models (89), and, indeed, as discussed below, UC and CD have been successfully treated by blocking antibodies to TNF.

In addition to IFN-γ and TNF, the IL-23/IL-17 axis is prominent in IBD; for example, Th17 cytokines are elevated in human IBD (52). In a model of innate gut inflammation driven by *Helicobacter hepaticus* infection in RAG^−/−^ mice, IL-23 instigates colitis and is produced by an innate lymphoid cell population, the ILC3 subset (19). In immunologically intact mice, Th17 cells also produce IL-22, a member of the IL-10 family of cytokines, which may protect against colitis. In mouse DSS-induced colitis, IL-22 delivery attenuated disease (166), while IL-22^−/−^ mice suffered greater weight loss compared with wild-type mice. Likewise, in a T-cell transfer model of colitis, transfer of IL-22^−/−^ T cells resulted in a more severe phenotype of colitis than in mice infused wild-type T cells (187). Innate lymphoid cell production of IL-22, stimulated through the prostaglandin pathway, is also required to maintain gut barrier integrity (39). While in human ulcerative colitis, IL-22^+^ T cells were linked to amelioration of symptoms (17), in Crohn’s disease, the expression of IL-22^+^ T cells within inflamed mucosa act to increase expression of inflammatory cytokines within subepithelial myofibroblasts, and so the role of IL-22 may be highly context dependent.

As type 2 immune cells (e.g., Th2 and M2 macrophages) drive contrasting responses to Th1 and Th17 cell phenotypes, they may be beneficial where the latter subsets mediate pathology. One route by which type 2 responses can counteract colitis is through the intestinal macrophage population, the largest of any tissue in the body (12). In mouse models of IBD, IL-4/IL-13 has been used to polarize macrophages to the M2 phenotype, and transferring these macrophages results in an ameliorated phenotype of colitis (31, 78). Tregs are also key mediators of protection against colitis, as their inclusion together with effector T cells results in protection against disease in the T-cell transfer model (122, 158).

The crucial role of Treg-associated cytokines is supported by the observation that TGF-β_1_-deficient mice develop multiorgan lymphoproliferative disease of the gut (94, 96) while, IL-10^−/−^ and IL-10R^−/−^ mice develop a spontaneous colitis (93, 157). Again, macrophages are implicated in pathogenesis, as when lacking IL-10R, they are intrinsically proinflammatory and cause spontaneous colitis in mice, while pediatric patients with mutations in the IL-10 receptor have more proinflammatory macrophages and an IBD-like phenotype (157, 190).

Anticytokine therapy is a key current treatment of IBD, with the use of anti-TNF antibodies, such as infliximab and adalimumab. The antibody ustekinumab, which acts against p40 (the common subunit of IL-23 and IL-12), may be useful in IBD because of its role in blocking the differentiation of naïve T cells to Th1 and Th17 cells; however, other anticytokine reagents show little effect or make disease worse (e.g., secukinumab: anti IL-17A antibody), implying individual cytokines may have proinflammatory and anti-inflammatory effects (128). Vedolizumab is a monoclonal antibody against α_4_β_7_-integrin and results in gut-specific anti-inflammatory activity (46, 85). SMAD7, an intracellular protein that blocks TGF-β signaling, can be targeted in vivo. Mongersen, an oral SMAD7 antisense oligonucleotide, upregulates anti-inflammatory TGF-β effects and also shows promising results in therapy of Crohn’s disease (119).

Newer approaches to treatment of IBD include a trial of Treg therapy (36). Peripheral blood Tregs were isolated from patients and expanded in vitro in the presence of ovalbumin, before reinfusion into the same individual; this resulted in a reduction in the Crohnʼs disease activity score but did not reach clinical significance (36). With growing interest in the immunomodulatory properties of helminth parasites, the use of helminths or their products has also attracted attention as a potential novel therapy, as outlined below.

## Modulation of IBD by Helminths and Their Products

As discussed above, epidemiological studies have indicated that populations with higher helminth parasite burdens suffer fewer immune inflammatory conditions, such as allergy (114) and inflammatory bowel disease, and Crohn’s disease is known to be less frequent in helminth-endemic countries (40). A substantial number of experimental animal models have also been used to show amelioration of colitic disease by helminth infections (Table 2), with studies encompassing all three of the helminth taxonomical groups: the cestodes, nematodes, and trematodes. Interestingly, reports from two different parasite models (with cestode and trematode infections) have implicated macrophage populations in helminth-generated protection against intestinal pathology (78, 160). Mechanistically, induction of IL-10 has been a recurrent theme in analyses of cytokine levels in helminth-infected mice (77) alongside a generalized switch from Th1 to Th2 cytokine production (169), while the helminth-induced expansion of Tregs that suppress colitis has also been demonstrated (66).

**Table 2.** Effects of helminth infection or exposure on intestinal inflammation

Model	Detail	Suppression	Reference
*Heligmosomoides polygyrus* (Nematoda)			
IL-10-deficient colitis	C57BL/6 piroxicam-induced	Histopathology, IFN-γ and IL-12	(42)
RAG transfer model	IL-10−/− T cells + piroxicam	Histopathology	(115)
TNBS colitis	C57BL/6 d14 infection, d4 colitis	Histopathology	(156)
TNBS colitis	BALB/c d10 infection, d4 colitis	Histopathology, IFN-γ and TNF	(169)
RAG transfer model	IL-10−/− T cells + piroxicam	Histopathology, IFN-γ and IL-17	(15, 65, 66)
OVA-specific colitis	OVA-specific T cells and oral OVA	Histopathology, IFN-γ, and IL-17	(95)
DSS colitis	BALB/c mice, up to 18 days	Weight loss and fecal blood	(37)
*Hymenolepis diminuta* (Cestoda)			
DNBS colitis	Infection 8 days before DNBS	Clinical score, histopathology and Myeloperoxidase, IL-10 dependent	(77)
DNBS colitis	Infection 8 days before DNBS	Clinical score, histopathology, and myeloperoxidase	(78)
DNBS colitis	Infection 8 days before DNBS	Protection IL-25 dependent	(142)
*Schistosoma japonicum* and *S. mansoni* (Trematoda)			
DSS colitis	*Sm* Infection 8 wk before DSS	Weight loss, colon shortening, disease activity index	(160)
TNBS colitis	Mice exposed to *Sm* eggs	Histopathology, IFN-γ, and mortality	(41)
TNBS colitis	Mice exposed to *Sj* eggs	Histopathology, IFN-γ	(117)
TNBS colitis	Mice exposed to *Sj* eggs (freeze-thawed)	Histopathology, IFN-γ, and bacterial translocation	(188)
TNBS colitis	Rats infected with *Sm* 7 days before TNBS	Histopathology and myelo-peroxidase	(120)
*Trichinella spiralis* (Nematoda)			
DNBS colitis	Infection 21 days before DNBS	Histopathology, IL-12 and myeloperoxidase	(84)
TNBS colitis	Infection 21 days after TNBS	Histopathology, myeloperoxidase and mortality	(189)

RAG, recombination activating gene; TNBS, trinitrobenzenesulfonic acid; OVA, ovalbumin; DSS, dextran sodium sulfate; DNBS, dinitrobenzene sulfonic acid.

Colitis can be induced in a number of animal models, in each of which, authors have demonstrated the effectiveness of helminth infections, or exposure to helminth eggs, in reducing disease severity scores, improving histological inflammation, and in dampening inflammatory cytokine profiles, such as IFN-γ and IL-17 (Table 2). The impact of different species in each model reflects the ability of helminths to promote chronicity of infection and immunological tolerance through a variety of mechanisms (113, 161).

One widely studied helminth model is the murine intestinal nematode *Heligmosomoides polygyrus* (144). In early studies, it was shown that the propensity of IL-10-deficient mice to develop colitis (exacerbated by administration of the nonsteroidal anti-inflammatory drug piroxicam) was ameliorated by *H. polygyrus* infection (42), and the same protective effect was observed when transferring IL-10-deficient T cells to RAG-deficient mice, which normally develop severe colitis (115). In more direct, and acute, models of colitis, it has been found that both BALB/c and C57BL/6 mice given infective *H. polygyrus* larvae orally showed reduced severity of trinitrobenzenesulfonic acid (TNBS) colitis (156, 169), and increased mucosal electrical resistance, indicating improved barrier function (156). In addition, the fourth-stage larvae of the same parasite improved disease score and histopathology in BALB/c mice suffering the effects of DSS-induced colitis (37).

The *H. polygyrus* model has also been very instructive at the mechanistic level. Foxp3^+^ Treg cells isolated from the mesenteric lymph node of *H. polygyrus*-infected mice were adoptively transferred into RAG^−/−^ mice and conferred protection from piroxicam-induced colitis, whereas Foxp3^+^ Treg cells from uninfected animals did not (15, 66); these data correlate with the known potency of *H. polygyrus* to activate the host Treg cell compartment (159). In addition, adoptive transfer of dendritic cells from *H. polygyrus*-treated mice in a RAG^−/−^ T-cell transfer model improved histological inflammation: these DCs were able to block ovalbumin (OVA)-induced cytokine secretion in vitro (15).

Other live helminth infections found to be protective include the rat cestode tapeworm *Hymenolepis diminuta*; mice infected with this parasite showed improved clinical scores and histopathology in a dinitrobenzene sulfonic acid (DNBS)-induced model of colitis (77, 78). Interesting mechanistic studies in this system have shown that protection required established infection, as STAT6-deficient mice both cleared the parasite and developed severe colitis (77); moreover, protection by infection was abolished by anti-IL-10 blocking antibodies (77). Protection was found to be mediated via the dominant population of alternatively activated macrophages (AAMs) generated by *H. diminuta* infection; macrophage depletion with clodronate-loaded liposomes reduced the effects of *H. diminuta*, while adoptive transfer of in vitro*-*generated AAMs was protective (78). Furthermore, protective myeloid cells could be generated in vivo by injection of *H. diminuta* antigens, with the resultant CDllb^+^F4/80^+^Ly6C^hi^Gr-1^lo^ population able to block DSS-induced colitis in recipient animals (143). A broader network of regulatory cells are, however, generated during this infection, such that splenic regulatory B cells can also confer protection against colitis (141), as well as dendritic cells pulsed with *H. diminuta* antigen were also successfully transferred to treat a DNBS colitis (108). Most recently, the protective effects of *H. diminuta* infection, and of the myeloid population induced by the parasite, have been shown to be inhibited by IL-22, but promoted by IL-25, with disease scores in DNBS-induced colitis exacerbated by anti-IL-25 antibody treatment (142).

In a similar manner, *Schistosoma mansoni* infections have also been shown to reduce the severity of experimental colitis in both DSS (160) and TNBS (120) models, again with involvement of the macrophage compartment (160). A number of investigators have also tested the ability of schistosome eggs, known to be potent immunomodulators, to influence colitis; eggs of both *S. mansoni* and a related species *S. japonicum* show protective effects, and Treg cells were found to be increased in spleens of *S. japonicum*-egg treated TNBS mice compared with TNBS alone (117). The exposure to *S. japonicum* eggs also resulted in reduced idiopathic bacterial transfer during TNBS colitis (188).

Finally, in another nematode infection system, *Trichinella spiralis* was also found to ameliorate both DNBS- and TNBS-induced colitis (84, 189) but not the type 2-mediated oxazolone colitis (189). Although few mechanistic insights into this system are as yet available, there is a clear indication of a cytokine switch, resulting from infection, with reduced IL-12 and higher levels of type 2 cytokines in infected mice challenged with the colitis model (84, 189).

## Human Therapy

Deliberate infection of humans with live parasites has already been tested for the potential to modulate these gut inflammatory diseases. In UC, a notable report was that from a single individual who self-medicated with *Trichuris trichiura*, the human whipworm (17). The patient’s symptoms resolved, and this was associated with increased IL-22 from T-helper cells, consistent with a protective effect for this cytokine, as discussed above. Experimental trials have also been performed with the hookworm *Necator americanus* in celiac disease patients (32, 33), whose clinical outcome demonstrated suppression of inflammatory cytokines (112). Infection also allowed patients with celiac disease to tolerate increasing gluten load and increased gut microbial richness (58).

The most widely used agent, however, has been the pig whipworm *Trichuris suis*, which was selected because it is short-lived in humans and minimally pathogenic (180). Administration of *T. suis* ova has been used successfully in small-scale trials to alleviate active CD and UC (44, 167, 168); however, two larger-scale trials, one including over 200 patients, were recently discontinued due to unusually high placebo response rates (44), and hence, the future of this approach has yet to be determined. A recent Cochrane review concluded that there is insufficient evidence to determine the safety and efficacy of helminth therapy for human IBD (53). Further randomized controlled trials are required to assess the efficacy of helminth infections as a treatment of inflammatory bowel disease.

A recent study on idiopathic chronic diarrhea in captive macaques also found alleviation of disease by deliberate helminth infection (18). Interestingly, this implied an increase in diversity of microbiota in association with *T. trichiura* infection. Potentially, the helminth infection restored intestinal diversity, an important cofactor to consider for future studies.

Currently, the landscape for live helminth therapy is uncertain; treatments have generally proven to be safe, but promising case reports and small-scale trials have not progressed successfully through large trials for a variety of logistical reasons, leaving us still short of an unequivocal randomized controlled study that would establish efficacy (44, 45).

## Molecular Approaches

Although there is strong evidence that live parasite infections exert profound down-modulatory effects on the immune system of their hosts, the therapeutic application of deliberate parasite infection is fraught with ethical and practical problems (45, 81). Hence, the use of defined molecular products from the same parasites is being explored as potential immunomodulators. A number of groups are testing parasite products in immunological disorders of the gastrointestinal tract (Table 3).

**Table 3.** Helminth products and proteins in intestinal inflammation

Molecules	Detail	Suppression	Reference
Nematode extracts and ES			
*Ancylostoma. caninum* ES	DSS colitis	Histopathology, cytokines, weight loss	(47)
*A. caninum* soluble proteins	TNBS colitis in Swiss mice	Histopathology, MPO	(150)
*Ancylostoma ceylanicum* extract, ES	DSS colitis in BALB/c mice	Histopathology, cytokines, MPO	(20)
*Trichinella spiralis* larval extract	DNBS colitis in C57BL/6 mice	Histopathology, MPO, IL-1β response; raised TGF-β, IL-13	(121)
*T. spiralis* ES	DSS colitis in C57BL/6 mice	Histopathology, disease activity, cytokines	(184)
Nematode proteins			
*Anisakis simplex* MIF homolog	DSS colitis in C57BL/6 mice	Disease activity index, weight loss	(23)
*Brugia malayi* asparaginyl-tRNA synthase	T-cell transfer model	Histopathology	(91)
*B. malayi* cystatin	DSS colitis in BALB/c mice	Disease activity score, histopathology	(85)
*B. malayi* ALT 2 protein	DSS colitis	Disease activity score, myeloperoxidase activity	(86)
*Toxascaris leonina* galectin	DSS colitis in C57BL/6 mice	Disease activity index, weight loss; raised TGF-β, IL-10	(87)
Trematode extracts			
*Schistosoma mansoni* soluble proteins	TNBS colitis in Swiss mice	Histopathology, MPO, IFNγ response	(150)
*S. mansoni* soluble extract	T-cell transfer model	Clinical disease score, colonoscopy, myeloperoxidase	(70)
Trematode proteins			
*Clonorchis sinensis* cystatin	DSS colitis in C57BL/6 mice	Disease activity index	(80)
*S. mansoni* 28-kDa glutathione S-transferase (P28GST)	TNBS colitis in rats	Reduced clinical and histological scores, 50% reduction in colonic MPO	(38)
*Schistosoma japonicum* cystatin	TNBS colitis in BALB/c mice	Histology, cytokine responses	(179)

ES, excitatory/secretory; MIF, migration inhibitory factor.

In earlier studies, parasite extracts or collections of excretory/secretory (ES) products were first tested for their protective effects against disease activity in a variety of mouse IBD models. Soluble extracts of the dog hookworm *Ancylostoma caninum* reduced clinical disease scores and abated the profile of inflammatory cytokines (IFN-γ, IL-17, and TNF) in both DSS- and TNBS-induced colitis models (20, 150). Likewise, both somatic extract and ES products from the closely related *A. ceylanicum* also suppressed DSS-induced colitis in mice (20), as did extract and ES from the pork nematode *Trichinella spiralis* (121, 184). Within the trematode models, soluble extracts of *S. mansoni* have protected mice against both TNBS-induced colitis (150), and in the T-cell transfer model into RAG-deficient hosts (70).

More recently, it has become possible to test individual defined products from helminth parasites, expressed as recombinant proteins; in principle, this approach should accelerate the translation from helminth infection to a molecular therapy for colitis. To date, however, only limited information has appeared, often lacking appropriate control proteins (such as inactive mutants, or even unrelated proteins expressed in the same recombinant vector). Nevertheless, it has been reported that *Brugia malayi* cytoplasmic asparaginyl-tRNA synthetase (BMAsnRS) improved colitis scores in a T-cell transfer model, an improvement attributed by the authors to the ability of BMAsnRS to bind IL-8 (91). Other *B. malayi* proteins linked to protection from colitis include ALT-2 (86), an abundantly expressed larval product previously shown to inhibit IFN-γ signaling (60) and CPI-2 or cystatin (85), which blocks antigen processing in mammalian cells (107). However, control inactive mutants of these proteins were not tested in the published reports.

Some studies have further explored the cellular mechanisms through which helminth products may protect from colitis. Similar to the parasites themselves, parasite-derived molecules predominantly stimulate a type 2 response in innate cells, as well as activate Tregs (Table 3). Innate immunity, in particular, plays an important role in ameliorating colitis severity, linked to IL-10 production. Interestingly, the macrophage migration inhibitory factor (MIF) homolog from *Anisakis simplex* (*As*-MIF) has also been shown to induce upregulation of IL-10 in both lymph node and intestinal epithelial cells, and also increases Foxp3^+^ Treg expression in mice subject to DSS-induced colitis (23). Returning to the cystatin family of inhibitors, a recombinant cystatin from *S. japonicum* (rSjcystatin)-induced Foxp3^+^ Treg cells and improved disease activity scores in TNBS-induced colitis (179), while a more distant homolog (CsStefin-1) from the liver fluke *Clonorchis sinenis* was shown to increase IL-10-positive macrophages in the DSS-induced colitis model (80).

In a similar vein, the galectin from the feline intestinal nematode *Toxascaris leonina*, provided modest protection against disease activity in DSS-induced colitis, while raising IL-10 and TGF-β responses (87), while a schistosome enzymatic protein, the 28-kDa glutathione-*S*-transferase, P28GST) conferred a protective effect that was dependent on eosinophil infiltration, as the effect was absent in IL5^−/−^ mice (38). Notably, each of the studies quoted here tested a single recombinant protein in the absence of controls that would exclude trivial immune deviation effects (from administration of an exogenous antigen) or potential contaminants introduced through the recombinant expression system.

## Conclusions and Outlook

Inflammatory bowel diseases have been treated with powerful immunosuppressive medications such as Infliximab, which severely dampens the body’s ability to mount a protective response in an infection. Helminths have existed symbiotically with humans for many millennia and have developed sophisticated means of manipulating the immune system to their advantage without greatly compromising antimicrobial defenses. The discovery that helminths and helminth-derived products can alleviate colitic disease in model systems may, thus, be key in deriving novel compounds that are effective against a range of autoimmune diseases, while maintaining the ability to fight bacterial infections.

## GRANTS

F. Varyani and R. M. Maizels gratefully acknowledge funding support from The Wellcome Trust, through the Edinburgh Clinical Academic Track (ECAT) and a Senior Investigator Award (Ref. 106122), respectively. J. O. Fleming gratefully acknowledges funding support from the National Multiple Sclerosis Society, USA (RG 3613A4/1). R. M. Maizels is also funded through the Wellcome Centre for Molecular Parasitology, supported by core funding from the Wellcome Trust (Ref. 104111).

## DISCLOSURES

No conflicts of interest, financial or otherwise, are declared by the authors.

## AUTHOR CONTRIBUTIONS

F.V., J.O.F., and R.M.M. drafted manuscript; F.V., J.O.F., and R.M.M. edited and revised manuscript; F.V., J.O.F., and R.M.M. approved final version of manuscript.
